# Supplementary material for: A qualitative analysis of the nurturing care environment of families participating in Brazil’s Criança Feliz early childhood program
Source: PLoS One. 2023 Jul 19;18(7):e0288940. doi: 10.1371/journal.pone.0288940 (PMC10355381; doi:10.1371/journal.pone.0288940)
Supplement: S1 Appendix — Customization and modification of TIDieR items were not applicable to this description. (DOCX) [file pone.0288940.s001.docx]

**S1 Appendix.** **Description of the Programa Criança Feliz (PCF) in Brasilia, Brazil: principles and governance***

| Name | Brazil’s Criança Feliz Program in the Federal District |
| --- | --- |
| Why | Integrated early childhood programs are essential to promote equity in early life. The PCF is a multisectoral nurturing care program targeting the most vulnerable communities, and its ultimate objective is to help reduce poverty, inequality, and violence. [1,2] |
| What | The PCF is based on the following initiatives:   1. Periodic home visits, by a trained professional, and complementary actions that support pregnant women and families to facilitate early childhood development; 2. Training and continuing education of professionals who work with pregnant women and children in early childhood, to improve service and strengthen the integrated performance of the network; 3. Development of content and supporting materials for intersectoral education of women, children in early childhood, and their families; 4. Support administrative regions of the Federal District, aiming at intersectoral mobilization and articulation, as well as the adequacy of infrastructure and services for program implementation; 5. Promote technical studies and scientific research about comprehensive childhood development; 6. Monitor and assess the actions, services, programs, and funds invested in early childhood in the Federal District, as well as monitor growth indicators and childhood development; 7. Periodic dissemination of information about the meaning of early childhood and about the results of good practices carried out aiming to form a culture that values early childhood in the DF, through the support and power of the government to determine the message.   PCF target audience is:   1. Pregnant women, children up to three years old, whose families are beneficiaries of the Bolsa Família Program; 2. Children up to six years old whose families are beneficiaries of the Continuous Cash Benefit (BPC – available to people 65 years old or older as well as to people of any age with disabilities); 3. Children up to six years of age removed from their families; 4. Pregnant women and young children who are in situations of extreme poverty, disability, or deprivation of the right to live with their family and community life; 5. Parents in prison deprived of liberty, deprived of traditional peoples or communities, among other situations of risk or vulnerability. [1,2,3] |
| Who | 1. The implementation of PCF is led by the Civil House within the Government Palace. 2. The Executive Secretary coordinates the PCF Multisectoral Committee, which articulates multisectoral actions and it is made up of the following agencies: I – DF Casa Civil, which coordinates it; II – The DF State Department for Social Development; III – The DF State Department for Health; IV – The DF State Department for Education; V – The DF State Department for Justice and Citizenship; VI – The DF State Department for Women; and VII – The DF State Department for Sports and Leisure. 3. The PCF is administered by the Department of Social Development (SEDES) where master trainers are located and are responsible for supervising, orienting, and promoting the continuing education of the supervisors and home visitors. 4. The Institute of Education, Sports, Culture, and Popular Arts (IECAP) (a non-profit organization) was contracted by SEDES to execute the PCF home visits. IECAP is responsible for hiring PCF staff - home visitors and supervisors. 5. Supervisors are responsible for monitoring and motivating the visitors. They are also responsible for holding individual weekly meetings with the team and for making a monthly report of all activities performed by their team. 6. Home visitors are responsible for conducting the intervention through home visits for up to 30 children or pregnant women. [1,2] |
| How | 1. The PCF services are implemented within the Social Assistance Reference Centers (CRAS), which are permanent units within the Unified Social Assistance System (SUAS) responsible for providing various social assistance services to meet social demands in a specific territory. 2. The PCF initial training for home visitors includes 80 hours of training, 40 hours of which are in the WHO/UNICEF Care for Child Development, and the remaining 40 hours consist of training in the home visit methodology. 3. The PCF relies on the support of the Multisectoral Early Childhood Centers to promote multisectoral coordination and care for children and pregnant persons in the territory. The Multisectoral Early Childhood Centers is coordinated by the social assistance sector and is made up of representatives from the (1) Social Assistance Reference Centers (CRAS), (2) Specialized Social Assistance Reference Centers (CREAS), (3) PCF Supervision in Brasilia, (4) Psychosocial Care Center (CAPS), (5) Extended Family Health Center (NASF), (6) Teaching Coordination, (7) Guardianship Council. The Multisectoral Early Childhood Centers are present in the following administrative regions: Ceilândia, Estrutural, Taguatinga, Riacho Fundo I, Riacho Fundo II, Samambaia, Recanto das Emas, Santa Maria, Paranoá, São Sebastião, Itapoã, Varjão, Brazlândia, Fercal, Sobradinho, and Planaltina. [1,2,4] |
| Where | The PCF was instituted in 2019 and implemented in eight administrative regions (Samambaia, Santa Maria, Recanto das Emas, Ceilândia, Riacho Fundo I, Riacho Fundo II, Estrutural, and Taguatinga), serving 1,600 individuals, in its first phase. Activities were suspended in 2020 with the onset of the COVID-19 pandemic. In 2021, the second phase of implementation began with the resumption of the PCF in the regions of the first phase and encompassing eight other administrative regions (Brazlândia, Paranoá, Varjão, Itapoã, Fercal, Sobradinho, Planaltina, and Gama) with the goal of serving 3,200 individuals. [2] |
| When and how much | The funds are transferred annually through the National Social Assistance Fund to cover training and technical assistance costs. The local government also provides funds to support PCF implementation. [5] |
| How well | In the Federal District (*Distrito Federal,* DF), the objectives of the PCF are to provide home visits aimed at promoting comprehensive early childhood development by adopting a multisectoral approach that articulates existing public policies to ensure comprehensive service, care, education, and assistance to children and their families as well as communities. The PCF uses the evidence-based WHO/UNICEF Care for Child methodology for training and conducting home visits. [1, 2] |
| *Customization and modification of TIDieR items were not applicable to this description. | |

**References**

1. Distrito Federal. Decreto 39867. Institui o Programa Criança Feliz Brasiliense e cria seu Comitê Gestor no âmbito do Distrito Federal. [Internet] Distrito Federal: Governo do Distrito Federal [cited 2023 Mar 15]. Available from: <https://www.sinj.df.gov.br/sinj/Norma/b4364404f7fd4837969fad2ad70f7e35/Decreto_39867_31_05_2019.html>
2. Buccini G, Godoi L, Gubert M. Análise da Implementação Municipal do Programa Criança Feliz: Relatório Técnico - Distrito Federal. University of Nevada Las Vegas (UNLV); Estados Unidos. Fevereiro, 2023.
3. Distrito Federal. Portaria 29 de 08 de desembro de 2020. Institui Núcleos Intersetoriais de Primeira Infância no Distrito Federal. [Internet]. Distrito Federal : Casa Civil [cited 2023 Mar 15]. Available from: <http://www.tc.df.gov.br/sinj/Norma/656f165d6a1d47488ad3d0c8d58dc2d8/Portaria_29_08_12_2020.html>
4. Brasil. PROGRAMA CRIANÇA FELIZ. A intersetorialidade na visita domiciliar. [Internet]. Brasilia, DF: Ministro do Desenvolvimento Social e Agrário; 2017 [cited 2023 Mar 15]. Available from: https://www.mds.gov.br/webarquivos/publicacao/crianca_feliz/A_intersetorialidade_na_visita_domiciliar_2.pdf
5. Brasil. Portaria n^o^ 2.496, de17 de setembro de 2018. Dispõe sobre o financiamento federal das ações do Programa Criança Feliz/Primeira Infância no SUAS, no âmbito do Sistema Único de Assistência Social, e dá outras providências. [Internet]. Brasil: Ministério do Desenvolvimento Social. 2018 [cited 2023 Mar 15]. Available from: <https://www.in.gov.br/materia/-/asset_publisher/Kujrw0TZC2Mb/content/id/41227841>
