# Supplementary material for: A qualitative analysis of the nurturing care environment of families participating in Brazil’s Criança Feliz early childhood program
Source: PLoS One. 2023 Jul 19;18(7):e0288940. doi: 10.1371/journal.pone.0288940 (PMC10355381; doi:10.1371/journal.pone.0288940)
Supplement: S2 Appendix — Translated from the original Portuguese version. (DOCX) [file pone.0288940.s002.docx]

**S2 Appendix. In-depth interview guide. Translated from the original Portuguese version.**

**DETAILED INTERVIEW GUIDE FOR KEY INFORMANTS OF THE PROGRAMA CRIANÇA FELIZ**

Hello, my name is ________. I am conducting interviews with families that participate in the Programa Criança Feliz to better understand how the program works in your municipality.

(Read consent form)

IDENTIFICATION:

| How many people do you live with? | |
| --- | --- |
| How old is your child? | |
| How long has your child been receiving the program? | |
| Is your child in school? | |
| Gender: | |
| Age: | |
| What is your skin color or race: | ( ) White  ( ) Black  ( ) Brown  ( ) Asian  ( ) Indian (native)  ( ) Did say or respond |
| Do you work (outside the home)? | ( ) Yes  ( ) No |
| Until what grade did you study? | ( ) Incomplete Elementary Education  ( ) Completed Elementary Education  ( ) Incomplete High School Education  ( ) Completed High School Education |
| Do you receive any social welfare benefits? What? (If yes, specify the reason) |  |

QUESTIONS:

**GENERAL**

1. For you, what is the Programa Criança Feliz?
2. Have you received guidance about your child’s development from CRAS? Have you received visitors? Online follow-up? *If you haven’t received a visit or follow-up: Would you like to?*

**HOME VISITS**

1. Does or has your child received visits in your home from the Programa Criança Feliz? *If it is online activities, ask: Tell me about the WhatsApp group?* What do you say? Who participates? What materials are used? Did you compile them? Were the activities difficult to do? Give me an example.
2. Regarding the last program visit you received, describe to me what happened from the time the visitor arrived until after they left. Is each visit different? Do the activities performed include other people in the house? What materials do you use in the activities?
3. Do you see as the benefits of the visits or follow-ups of your child and your family? If so, could you give some examples?
4. What do you think about the frequency and duration of the visits or follow-up? Is it always the same person doing the visiting or follow-up?
5. How did you hear about the program? When did you start receiving visits and follow-ups?

**MULTISECTORAL ACTIONS**

1. Have you ever been referred to any service through the Programa Criança Feliz? If yes, for which service? In what situation and why?
2. Is your child followed up at the UBS (Primary Healthcare Center)? How often? (For example, prenatal care, vaccination). At the UBS, do they discuss your child’s development? Has your child ever had any health problems? When and how did you find help?
3. Did you receive home visits after the birth of your child? What were they like? Until when did you receive them?
4. Is your child in daycare?
5. *If the daycare sends online activities or homework assignments: What are the activities like?* Did you have any difficulties? Did you have difficulty accessing the internet to get the activities?
6. Have you participated in other programs or public services? If yes, which ones? How did they work?
7. Besides the visits, has the Programa Criança Feliz helped your family in other ways? (For example: *Healthcare, Social assistance, Education)*

**FAMILIES’ NEEDS**

1. How do you think your child is developing? Are you worried about your child’s development? If so, have you sought help? Where?
2. How is your relationship with your child? Do you play with him/her? Does he/she have toys? Which game do you most like or do together? Which game do you think helps his/her development the most? Do you read to your child? Watch television?
3. Have you ever had difficulty dealing with your child’s behaviors? For example, saying no, excessive crying, and tantrums. If so, what did you do?
4. Do you feel emotionally overwhelmed? If so, is this overload related to motherhood?
5. Do you have space in your home to play with your child? Do you pay rent? In your home, have you ever had problems with the water supply? Sewage? Electricity?
6. What were your expectations regarding the Programa Criança Feliz? Has the PCF offered everything you want? Are there any expectations that the program hasn’t met? Provide examples.

**COVID-19**

1. How has the pandemic changed your family? For example, if the child attended daycare, how did you deal with the closing of the schools? How was it to stay home with the child? (Use of punitive disciplines, for example). Has the child stopped going to the UBS, stopped taking vaccinations?

**FOOD INSECURITY**

1. *If you receive a food basket: How often?* Is it sufficient to meet your family’s needs? *Follow-up with the Food Insecurity questions.*
2. Before I go on to the final questions, I would like to know more about the food situation of the people who live in your home. Answer yes or no to the next two questions:

- “In the past 12 months the food that (I/we) bought just didn’t last, and (I/we) didn’t have money to get more” ( ) Yes ( ) No [1, 2]
- “In the past 12 months, did (you/you or other adults in your household) ever cut the size of your meals or skip meals because there wasn't enough money for food?” ( ) Yes ( ) No [1, 2]
- If yes for both, do you believe that the Programa Criança Feliz or some other government program could help you with this situation?
- If yes, how have you dealt with it?

1. Do you breastfeed? How long did you breastfeed? Why?

**SUSTAINABILITY**

1. Would you change anything in the program?
2. How do you feel about continuing in the program?
3. Is there anything else you would like to share about your experience with the program?

Final thanks. Stop recording.

**References**

1. Hager ER, Quigg AM, Black MM, et al. Development and validity of a 2-item screen to identify families at risk for food insecurity. Pediatrics. 2010;126(1):e26-e32. doi:10.1542/peds.2009-3146
2. Poblacion A, Segall-Corrêa AM, Cook J, de Aguiar Carrazedo Taddei JA. Validity of a 2-item screening tool to identify families at risk for food insecurity in Brazil. Cad Saude Publica. 2021;37(6).
