# Supplementary material for: A qualitative analysis of the nurturing care environment of families participating in Brazil’s Criança Feliz early childhood program
Source: PLoS One. 2023 Jul 19;18(7):e0288940. doi: 10.1371/journal.pone.0288940 (PMC10355381; doi:10.1371/journal.pone.0288940)
Supplement: S3 Appendix — (DOCX) [file pone.0288940.s003.docx]

**S3 Appendix. Themes and subthemes identified in interviews with families participating in the Programa Criança Feliz in the Federal District, Brazil.**

| **Themes** | **Subthemes** | **Brief description** |
| --- | --- | --- |
| **Good Health** | Child mental health | Caregivers perceived that PCF actions generated care and protection for the child’s mental health. |
|  | Maternal mental health | Caregivers reported that motherhood led to feelings of exhaustion, loneliness, anxiety, depression, and criticism. However, they normalize these feelings as well as being overtired. |
|  | Prior link with the healthcare system | Caregivers monitored the child’s growth and maternal/child well-being. |
| **Adequate Nutrition** | Household Food Insecurity | Caregivers were concerned about providing food for their children and reported insufficient quantity and quality of food. |
|  |  | Caregivers reported to the home visitors their need for food. |
|  | Breastfeeding | Families reported aspects related to early weaning or not breastfeeding. |
| **Responsive Care** | Family’s bond with the home visitor | Caregivers reported establishing a trusting relationship with their home visitors.  Families recognized the importance and value of the activities prepared by the home visitors. |
|  | Home visits | Caregivers reported that home visits are consistent and flexible to their families’ availability and needs.  Caregivers reported repeating the early stimulation activities after each completed home visit. |
|  |  | Barriers: Lack of materials to perform the activities and no interaction of caregivers with children during the activities. |
|  | Early Childhood Development (ECD) | Caregivers recognized that PCF activities promote ECD. |
|  |  | Barrier: Caregivers’ lack of information about what is expected for each developmental stage of their children. |
|  | Positive Parenting | Caregivers have learned to observe their children and recognize the importance of playing and reading books to promote early stimulation.  Pregnant persons learned how to interact with their baby while still in the womb. |
|  | Paternal participation | Caregivers reported little paternal participation in PCF activities and in the daily life of the family. |
|  | Responsive disciplinary practices | Caregivers learned that the way they deal with their children can influence their future and ECD. |
|  |  | Barriers: The PCF methodology does not provide the necessary support to caregivers about the use of responsive disciplinary practices. |
| **Opportunities for Early Learning** | Adequacy of early stimulation activities | Caregivers reported that home visitors performed activities according to their children’s interests and provided guidance to caregivers about substituting materials. |
|  | Perceived early learning | Caregivers recognized that the activities proposed by home visitors promote early learning. |
|  |  | Barriers: Caregivers reported the use of screen time such as educational cartoons on TV and cell phones as resources to help in calming the child. |
|  | Families’ appreciation of the PCF | Families reported the willingness to continue participating in the PCF.  Caregivers reported appreciation for the PCF. Receiving PCF generated feelings of welcoming and importance. |
|  | Families’ socioeconomic status | Barrier: Most of the caregivers are unemployed and have no income. |
|  | Secure environments | Caregivers reported efforts to make the community a safe environment for their children. |
|  |  | Barrier: Caregivers reported difficulties with access to basic sanitation, energy, and gas, along with stressful times. |
|  | Multisectoral actions carried out by the PCF | Caregivers reported receiving information about social rights and facilitating access to social welfare programs, which strengthened families’ bond with the social assistance sector. |
|  |  | Barrier: Caregivers reported that home visitors/PCF failure to execute multisectoral actions. |
|  | Social Support | Caregivers noted that home visitors extended care to the other children at home. |
|  |  | Caregivers’ participation in the conditional cash transfer programs (CCTPs) was facilitated by the PCF. |
|  |  | Caregivers reported the existence of a non-governmental support network to help their families. |
|  |  | Barriers: Caregivers reported that the CCTPs was a temporary benefit and not targeted to all PCF families. |
|  |  | Caregivers reported misleading promise of a financial benefit associated with the PCF. |
